# P-1427. Trends in Vaccination at Pharmacies Among Medicaid Recipients: A Claims Analysis

**DOI:** 10.1093/ofid/ofaf695.1614

**Published:** 2026-01-11

**Authors:** Amanda Eiden, Yi Zheng, Dong Wang, Marcie Fisher-Borne

**Affiliations:** Merck, Philadelphia, PA; Merck & Co., Inc., Boston, Massachusetts; Merck & Co., Inc., Boston, Massachusetts; Merck & Co., Inc.,, North Wales, Pennsylvania

## Abstract

**Background:**

Medicaid beneficiaries often have lower vaccination rates, partly due to limited access and challenges with traditional vaccine delivery settings. Pharmacies offer a convenient and accessible option for vaccinations in the US. Given the evolving immunization landscape during and after the COVID-19 pandemic and the unique vaccination behaviors of those with Medicaid, this research aimed to assess trends in pharmacy-based vaccination among adolescent and adult Medicaid recipients in the United States from 2018 to 2022.

**Figure 1.**
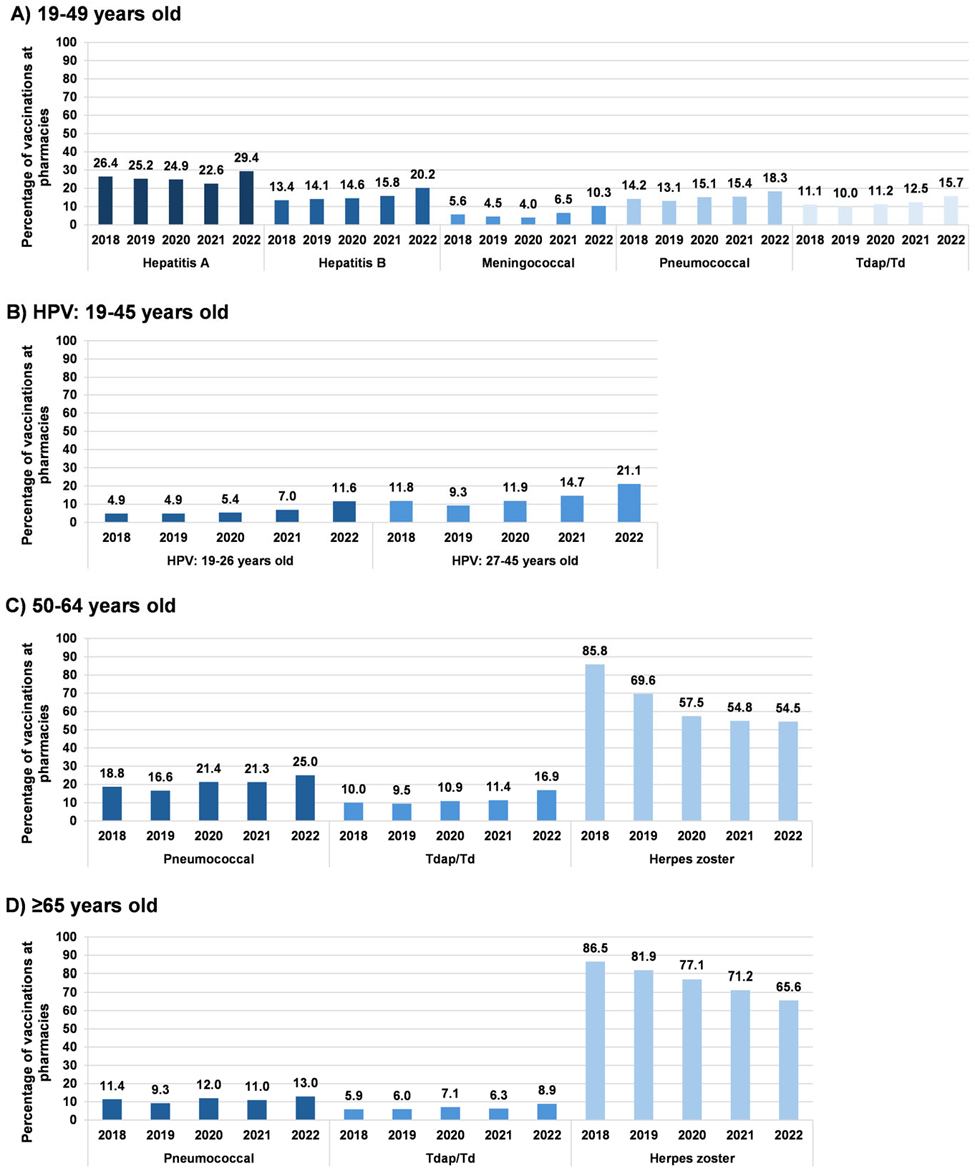
Percentage of routine vaccinations administered at pharmacies HPV, human papillomavirus; Td, combined tetanus and diphtheria; Tdap, combined tetanus, diphtheria, and acellular pertussis Bars indicate the percentage of all vaccinations in a calendar year that occurred in pharmacies.

**Figure 2.**
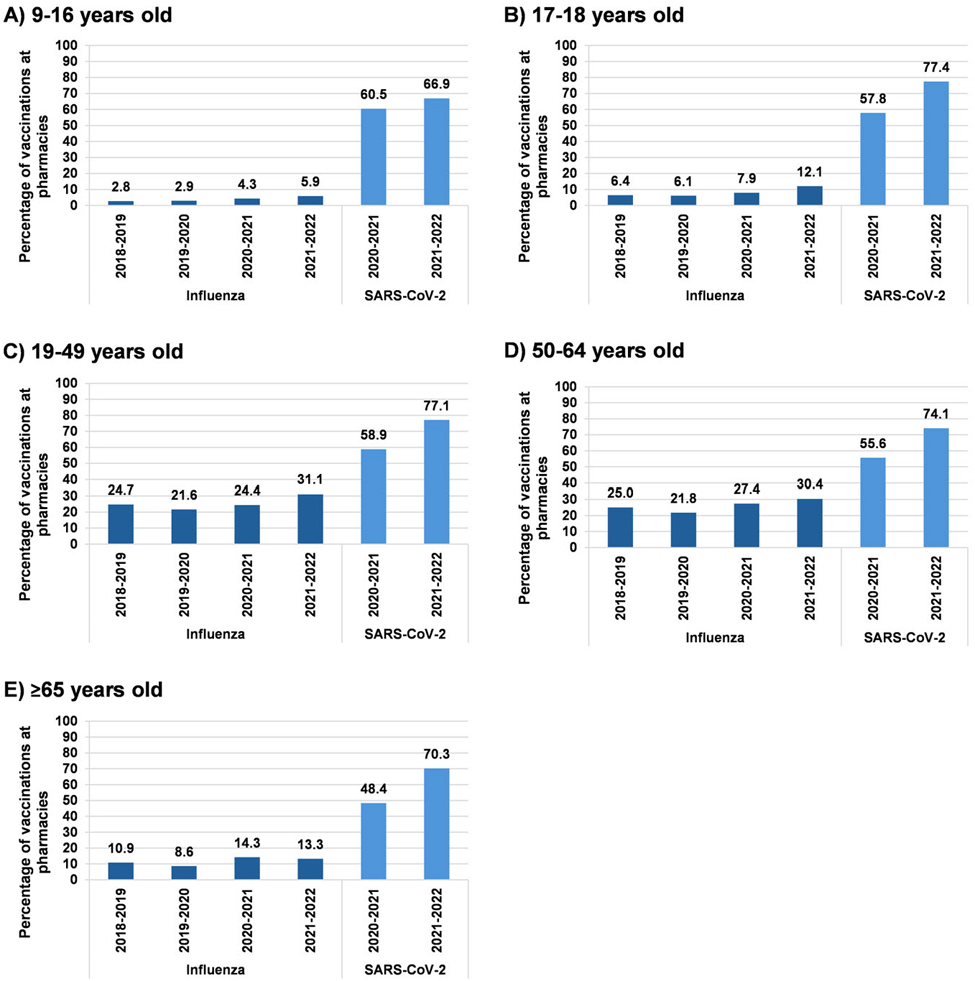
Percentage of seasonal vaccinations administered at pharmacies SARS-CoV-2, severe acute respiratory syndrome coronavirus 2

Bars indicate the percentage of all vaccinations in a given season occurring in pharmacies. Seasons ran from August 1 to July 31 of the following year. SARS-CoV-2 vaccines became available in December 2020, so these data for 2020-2021 represent a partial season.

**Methods:**

A retrospective analysis was conducted to assess trends in pharmacy-based vaccination among adolescent and adult Medicaid recipients from January 1, 2018 (pre-pandemic) to December 31, 2022 (peri-/post-pandemic). The percentage of all vaccinations administered at pharmacies was calculated, along with the overall percentage change in vaccination rates during the study period.

**Results:**

The overall routine and seasonal vaccine administration rates decreased after the pandemic, with a few exceptions. Among 9-16-year-olds, the percentage of routine vaccinations administered at pharmacies remained relatively stable and ≤0.8% during the study period. In contrast, adults aged 19-49 saw increases from 3.0 to 6.8 percentage points in routine vaccinations occurring at pharmacies for hepatitis A, hepatitis B, human papillomavirus (HPV), meningococcal, pneumococcal, and tetanus-containing vaccines. Among adults aged 50-64 and ≥65, receipt of pharmacy-based pneumococcal and tetanus-containing vaccines increased, while herpes zoster vaccination at pharmacies decreased. Across all age groups, the percentage of influenza vaccinations administered at pharmacies increased over the study period.

**Conclusion:**

Although overall routine and seasonal vaccination rates declined among adolescent and adult Medicaid beneficiaries during the COVID-19 pandemic, the percentage of vaccinations administered at pharmacies increased during and after this period, with the greatest proportion observed in 2022. These findings suggest that, as public health policies and delivery systems continue to evolve, pharmacies will play an ongoing role in improving vaccination coverage among under-resourced populations.

**Disclosures:**

Amanda Eiden, PhD, MBA, MPH, Merck & Co., Inc.: Stocks/Bonds (Public Company) Yi Zheng, PhD, MPH, Merck & Co., Inc.: Stocks/Bonds (Public Company) Dong Wang, PhD, Merck & Co., Inc.: Stocks/Bonds (Public Company) Marcie Fisher-Borne, PhD, Merck & Co., Inc.: Stocks/Bonds (Public Company)

